# The benefit of secondary prevention with oat fiber in reducing future cardiovascular event among CAD patients after coronary intervention

**DOI:** 10.1038/s41598-019-39310-2

**Published:** 2019-02-28

**Authors:** Jia-Ru Wu, Hsin-Bang Leu, Wei-Hsian Yin, Wei-Kung Tseng, Yen-Wen Wu, Tsung-Hsien Lin, Hung-I Yeh, Kuan-Cheng Chang, Ji-Hung Wang, Chau-Chung Wu, Jaw-Wen Chen

**Affiliations:** 1 Divison of Cardiology, Department of Medicine, Taipei Veterans General Hospital, Taipei, Taiwan; 2 Heath Care and Management Center, Taipei Veterans General Hospital, Taipei, Taiwan; 3 Cardiovascular Research Center, School of Medicine, National Yang-Ming University, Taipei, Taiwan; 4 Division of Cardiology, Heart Center, Cheng-Hsin General Hospital, and School of Medicine, National Yang-Ming University, Taipei, Taiwan; 5 Department of Medical Imaging and Radiological Sciences, I-Shou University and Division of Cardiology, Department of Internal Medicine, E-Da Hospital, Kaohsiung, Taiwan; 6 Cardiology Division of Cardiovascular Medical Center and Department of Nuclear Medicine, Far Eastern Memorial Hospital, New Taipei City, Taiwan; 7 Division of Cardiology, Department of Internal Medicine, Kaohsiung Medical University Hospital and Kaohsiung Medical University, Kaohsiung, Taiwan; 8 Mackay Memorial Hospital, Mackay Medical College, New Taipei City, Taiwan; 9 Division of Cardiology, Department of Internal Medicine, China Medical University Hospital, Taichung, Taiwan; 10 Graduate Institute of Clinical Medical Science, China Medical University, Taichung, Taiwan; 11 Department of Cardiology, Buddhist Tzu-Chi General Hospital, Tzu-Chi University, Hualien, Taiwan; 12 Division of Cardiology, Department of Internal Medicine, National Taiwan University College of Medicine and Hospital, Taipei, Taiwan; 13 Department of Primary Care Medicine, College of Medicine, National Taiwan University, Taipei, Taiwan; 14 Institute of Pharmacology, National Yang-Ming University, Taipei, Taiwan

## Abstract

There is limited information about the association between oat fiber intake and future cardiovascular events in CAD patients after coronary intervention for secondary prevention. This study enrolled 716 patients after coronary intervention in clinical stable status from the CAD cohort biosignature study. Patients were analyzed according to whether the presence of regular oat fiber intake during the follow-up period, and the association with endpoints including cardiovascular death, non-fatal myocardial infarction, non-fatal stroke and revascularization procedures were analyzed. The average follow-up period is 26.75 ± 8.11 months. Patients taking oat fiber were found to have lower serum levels of LDL, triglycerides, ratio of TC/HDL, as well as lower inflammatory markers values. After adjusting for confounders in the proportional hazard Cox model, oat fiber intake was associated with a lower risk of future revascularization (HR = 0.54, 95% CI 0.35–0.85; p = 0.007), and lower risk of major adverse cardiovascular events (HR = 0.62, 95% CI 0.43–0.88; p = 0.008), suggesting the association of oat fiber use and lower risk of future adverse event in CAD patients after coronary intervention.

## Introduction

Coronary artery disease (CAD) is associated with increased risk of morbidity and mortality, and is a leading cause of death worldwide. Patients with established CAD are recommended to receive lifestyle modification and optimal medical treatment in order to reduce future risk of developing adverse events.

Although there was no consistent association between dietary cholesterol and CVD risk and current guideline removed the limits on dietary cholesterol^1^, serum LDL cholesterol still contributed to plaque formation and was seen as the major cardiovascular risk factor. Therefore, healthy diet pattern emphasizes more vegetables, fruits, whole grains and low fat foods to patients at risk. Additionally, the newest dietary guidelines still advised to limit intake of saturated fats and trans fats, both of which can raise serum LDL cholesterol and was considered harmful for cardiovascular healthiness^1^. Increased intake of whole grains, especially oat fiber, has been reported to be beneficial for cardiovascular system, and to reduce the risk of cardiovascular disease (CVD)^2^. Oat β-glucan (OBG), the main soluble fiber found in oats, has a cholesterol-lowering effect^3,4^. To our interest, the association of oat fiber intake and future adverse event risk has not been reported in CAD patients who received coronary intervention and for secondary prevention. Our current study aimed to investigate the association of oat fiber intake and the risk of future CV events in CAD patients after coronary intervention.

## Methods

### Study population

The Biosignature study was a nationwide prospective cohort study to search for predictive marker among CVD patients in stable condition^5^. CAD patients was evaluated in 9 different medical centers located in Northern, Central, Southern, and Eastern Taiwan. CAD was diagnosed according to documented coronary angiogram, a history of myocardial infarction, or angina with ischemic ECG changes or positive stress test results. Patients were enrolled only if (1) they had received successful percutaneous coronary intervention (PCI) and (2) they had been stable on medical treatment for at least 1 month before enrollment as previously reported^5^. CAD patients who had dietary information about oatmeal intake were enrolled in this study. The study complied with the Declaration of Helsinki, which was approved by the appropriate Health Authorities, independent Ethics Committees in each hospital including Taipei Veterans General Hospital, Taipei Cheng-Hsin General Hospital, E-Da Hospital, Kaohsiung, Far Eastern Memorial Hospital, New Taipei City, Kaohsiung Medical University Hospital, Mackay Memorial Hospital, China Medical University Hospital, Taichung, Buddhist Tzu-Chi General Hospital, and National Taiwan University College of Medicine and Hospital. All patients gave their written inform consent before enrollment.

### Baseline data and oat intake

After enrollment, data were collected by trained study nurses and qualified cardiologists. Baseline characteristics including history of hypertension, diabetes, and smoking were recorded. Medications information and dosage was collected by chart review and structured questionnaires. Healthy diet was suggested to follow recommendation^6^ and defined as one containing more fruits, vegetables, nuts, reduced-fat dairy products, whole grains, and fish. Because it has been established that the consumption of at least 3 g per day of oat β-glucan can achieve a reduction in LDL cholesterol of up to 10% and reduce the risk of CVD by as much as 20%^7^, we used this as cutoff value to define oat intake. This amount is provided by approximately 55 g oat bran (minimum 5.5% β-glucan) or 75 g rolled oats (β-glucan)^8^ and this can be achieved through eating 2–4 portions of oat based products e.g. breakfast cereals, breads and crackers every day. Patient who adhered oat fiber intake during follow-up period more than 50% was considered oat fiber user. After enrollment, 20 mL of blood from peripheral vessels, and 10 mL urine were collected. Samples were stored at −80 °C until further analysis for the biomarkers study. Patients who had ingested any drugs with antioxidant activity, vitamins, or food additives within 4 weeks prior to blood/urine sampling were excluded.

### Clinical follow up for adverse cardiovascular events

All study patients who were initially stable under medical treatment were prospectively and regularly followed up at the individual hospital clinics. Primary endpoint is major cardiovascular events including cardiovascular death, non-fatal myocardial infarction, non-fatal stroke and revascularization procedures including coronary intervention and bypass surgery. The protocol for CV event follow-up was similar to that previously reported^5,9,10^.

### Statistics

The baseline characteristics of subjects in the oat fiber intake and without oat fiber groups were compared. The development of clinical adverse outcomes including non-fatal stroke, non-fatal myocardial infarction, repeat revascularization, and total CV events during follow-up period were compared between groups. Comparisons of continuous variables between groups were performed by ANOVA test, while subgroup comparisons of categorical variables were assessed by χ^2^ or Fisher’s exact test. The primary and secondary outcomes were described by an overall percentage and expressed by means of proportions with a 95% confidence interval (CI). Event-free survival rate was calculated using the Kaplan–Meier method, with the significance evaluation using log rank tests.

We tested the proportionality of hazards with the use of time-varying covariates. When proportional hazards could be assumed, oat fiber effects were estimated from Cox regression models to adjust for age, sex, and baseline risk factors. Independent baseline variables with a p-value of <0.05 in the univariate analyses and proposed associated confounders, including age, gender, smoking habit, history of hypertension, diabetes, lipid profiles, medication information as well as inflammatory markers including high sensitivity C-reactive protein (hs-CRP), and TNF-α were included in the multivariate analyses. The two-tailed alpha significance level in all the tests was 0.05.

### Ethics approval and consent to participate

The study complied with the Declaration of Helsinki, which was approved by the independent Ethics Committees and Review Boards in each hospital. All patients should give their written inform consent before enrollment.

## Results

A total of 1663 patients who underwent coronary intervention were screened and 716 patients who had dietary information at beginning and follow-up period were enrolled in this study. The oat fiber intake group included 242 patients (33.8%) and the demographic parameters of study subjects are listed in Table 1. Patients in the oat fiber intake group were older (68.82 ± 11.65 years vs. 65.26 ± 12.19 years, p < 0.01) compared to the no oat fiber group. The oat fiber intake group had a lower proportion of smokers (48.35% vs. 56.75%, p = 0.03), and a lower ratio of waist to hip circumference (0.94 ± 0.07 vs. 0.95 ± 0.08, p = 0.0067). However, there were no significant differences in baseline coronary severity and medications between the two groups. Patients in the oat intake had lower baseline serum LDL (92.84 ± 29.79 mg/dL vs. 97.91 ± 31.01 mg/dL, p = 0.04), serum triglycerides (121.86 ± 66.79 vs. 134.43 ± 86.44 mg/dL, p = 0.032) and TC/ HDL ratio (3.89 ± 1.13 vs. 4.08 ± 1.22, p = 0.047) compared to the no oat group, but there were no significant differences in the level of HDLs or glucose between the two groups (Table 2). Patients in the oat fiber intake group had lower serum levels of inflammation markers, including HsCRP (0.29 ± 0.55 mg/L *vs*. 0.43 ± 1.14 mg/L, p = 0.025) and TNF-α (4.06 ± 3.81 pg/ml *vs*. 4.99 ± 5.99 pg/ml, p = 0.012) compared to the no oat fiber group.

**Table 1:** Baseline Characteristics and Medication Use of the Study Population.

	Oat used		*p* Value
	No (n = 474)	Yes (n = 242)	
Age, years	65.26 ± 12.19	68.82 ± 11.65	0.0002
Male, n (%)	408 (86.08)	202 (83.47%)	0.353
Hypertension, n (%)	306 (64.56)	160 (66.12%)	0.679
Diabetes, n (%)	178 (37.55)	86 (35.54%)	0.597
Smoking, n (%)	269 (56.75)	117 (48.35%)	0.033
History of CAD, n (%)	117 (24.68)	60 (24.79%)	0.974
History of stroke, n (%)	11 (2.32)	5 (2.07%)	0.827
BMI, kg/m^2^	26.47 ± 5.24	25.92 ± 3.65	0.1005
Waist, cm	94.21 ± 10.23	93.34 ± 9.07	0.2438
Buttocks, cm	99.11 ± 8.43	99.9 ± 9.28	0.2523
Waist to hip ratio	0.95 ± 0.08	0.94 ± 0.07	0.0067
Systolic BP, mmHg	129.58 ± 17.93	129.3 ± 15.53	0.8297
Diastolic BP, mmHg	74.01 ± 12.36	72.58 ± 12.14	0.1394
Stenosis vessel, n (%)	2.01 ± 0.92	2.03 ± 0.94	0.8468
Stenosis lesion, n (%)	3.31 ± 2.21	3.27 ± 2	0.8382
Stenting, n (%)	1.56 ± 0.93	1.62 ± 0.87	0.4966
LVEF (%)	57.05 ± 12.36	56.17 ± 12.74	0.483
**Medication**			
Anti-platelet, n (%)	427 (90.0)	226 (93.4)	0.14
Aspirin, n (%)	345 (72.7)	188 (77.6)	0.155
clopidogrel, n (%)	223 (47.0)	117 (48.3)	0.742
Anticoagulant, n (%)	11 (2.3)	8 (3.3)	0.438
ACE inhibitor, n (%)	106 (22.3)	55 (22.7)	0.912
ARB, n (%)	208 (43.8)	106 (43.8)	0.984
Beta-blocker, n (%)	285 (60.1)	137 (56.6)	0.366
CCB, n (%)	211 (44.5)	112 (46.2)	0.653
CCB, DHP, n (%)	163 (34.3)	94 (38.8)	0.240
Diuretics, n (%)	62 (13.0)	29 (11.9)	0.677
Nitrate, n (%)	233 (49.1)	128 (52.8)	0.344
Statin, n (%)	333 (70.2)	172 (71.1)	0.820
PPIs, n (%)	20 (4.2)	10 (4.1)	0.956

Values are mean ± SD, or n(%). BMI indicates body mass index; LVEF, left ventricle ejection fraction; CCB, calcium channel blocker; DHP, Dihydropyridine; ACE = angiotensin converting enzyme; ARB, angiotensin II receptor blocker; PPIs, proton pump inhibitors.

**Table 2:** Biochemical profiles of study population.

	Oat used		*p* Value
	No (n = 474)	Yes (n = 242)	
Glucose, mg/dL	121.88 ± 47.38	118.14 ± 42.74	0.304
Cholesterol, mg/dL	162.03 ± 34.3	159.23 ± 33.89	0.299
LDL-cholesterol, mg/dL	97.91 ± 31.01	92.84 ± 29.79	0.044
HDL-cholesterol, mg/dL	41.99 ± 11.27	42.64 ± 10.48	0.455
Triglyceride, mg/dL	134.43 ± 86.44	121.86 ± 66.79	0.032
TC/ HDL ratio	4.08 ± 1.22	3.89 ± 1.13	0.047
Albumin, g/dL	3.82 ± 0.47	3.83 ± 0.49	0.794
eGFR, mL/min/1.73 m^2^	73.53 ± 29.6	76.32 ± 38.96	0.331
hsCRP, mg/L	0.16 (0.02–13.08)	0.14 (0.02–5.9)	0.025
TNF-α, pg/mL	3.03 (0.11–48.72)	2.65 (0.11–23.21)	0.012

Values are mean ± SD. LDL indicates low-density lipoprotein; HDL, high-density lipoprotein; TC, total cholesterol; eGFR, estimated glomerular filtration rate; hsCRP, High sensitivity C-reactive protein; TNF-α, tumor necrosis factor-α.

**Figure 1:**
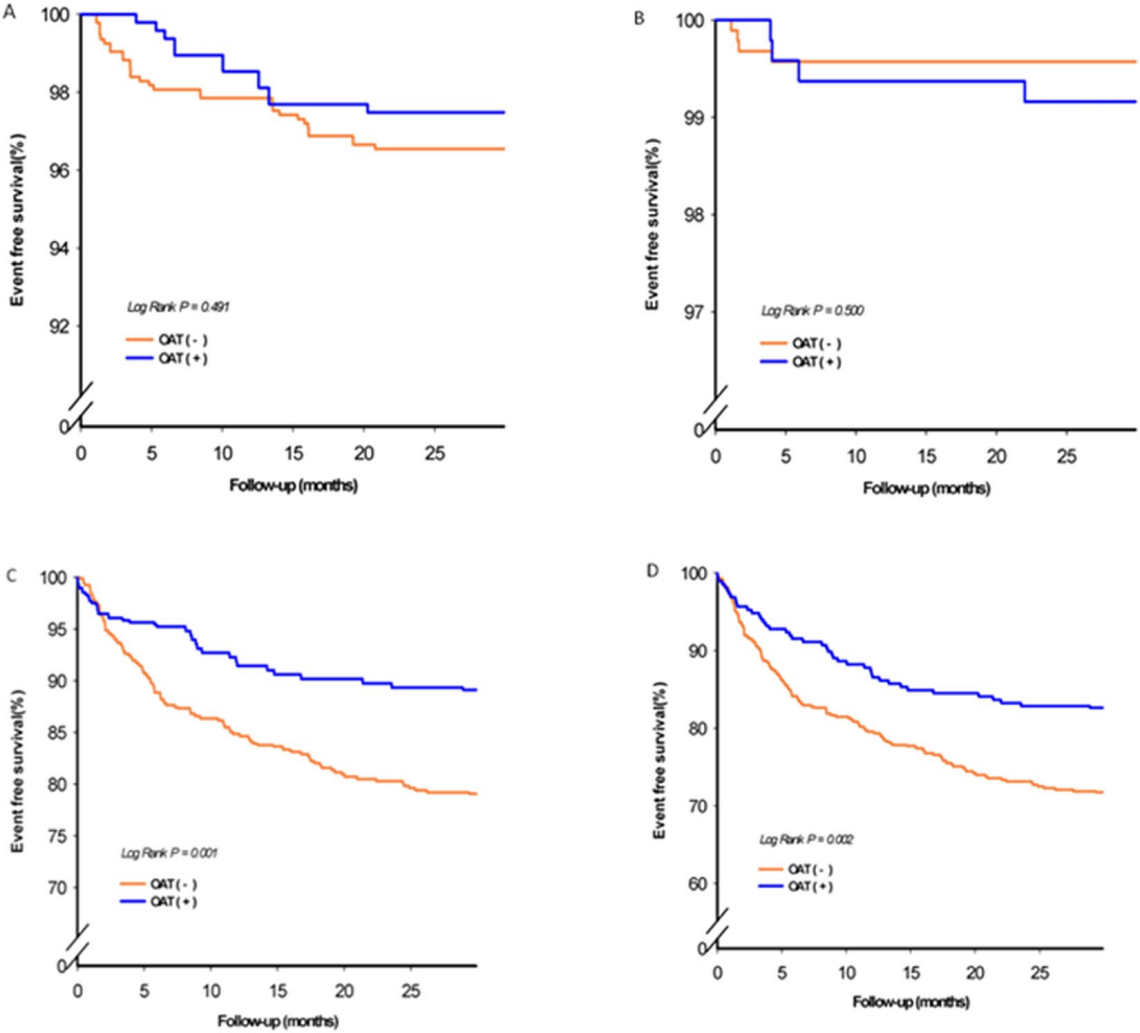
Kaplan-Meier estimates of survival free of cardiovascular events, including (**A**) myocardial infarction; (**B**) stroke, (**C**) revascularization treatment and (**D**) total cardiovascular event in subjects categorized according to whether oat is used.

During the average follow-up period of 26.75 ± 8.11 months, 175 patients (24.4%) experienced at least one element of the combined events of cardiovascular death (n = 12 [1.67%]), MI (n = 22[3%]), stroke (n = 4[0.5%]), heart failure (n = 14[1.9%]), or revascularization procedures including PCI or CABG (n = 123[17.2%]) (Table 3). Fig. 1 shows the results of the log-rank test and Kaplan-Meier survival analysis. Oat intake was associated with a lower risk of total CV events (p = 0.020 by log-rank test) and revascularization (p = 0.001 by log-rank test). After adjustment for confounders including sex, age, hypertension, diabetes, cigarette smoking, waist/hip ratio, hsCRP and TNF-α, the Cox proportional hazard regression model analysis showed that oat fiber intake was associated with 46% risk reduction in revascularization (HR = 0.54, 95% CI 0.35–0.85; p = 0.007) and 38% reduction in risk of total cardiovascular events (HR = 0.62, 95% CI 0.43–0.88; p = 0.008) (Table 3). There were no significant differences in the incidence of MI, stroke, and HF and CV death.

**Table 3:** Oat use and future risk of cardiovascular disease in CAD patients.

	Oat use		Model 1	p	Model 2	p	Model3	p
	No (n = 474)	Yes (n = 242)	HR (95% CI)		HR (95% CI)		HR (95% CI)	
MI, n(%)	16 (3.3)	6 (2.4)	0.72 (0.28 1–0.84)	0.493	0.76 (0.29–1.96)	0.564	0.83 (0.31–2.19)	0.702
Stroke, n(%)	2 (0.4)	2 (0.8)	1.94 (0.27–13.76)	0.508	2.26 (0.30–17.23)	0.433	2.14 (0.27–16.95)	0.470
CHF, n(%)	10 (2.1)	4 (1.6)	0.78 (0.24–2.47)	0.668	0.67 (0.20–2.20)	0.505	0.73 (0.22–2.42)	0.506
Revascularization, n(%)	97 (20.4)	26 (10.7)	0.49 (0.32–0.76)	0.001	0.52 (0.33–0.80)	0.003	0.54 (0.35–0.85)	0.007
CV death, n(%)	8 (1.6)	4 (1.6)	0.97 (0.29–3.23)	0.963	0.71 (0.21–2.38)	0.578	0.82 (0.24–2.89)	0.762
Total CV event, n(%)	133 (28.0)	42 (17.3)	0.58 (0.41–0.81)	0.002	0.58 (0.41–0.82)	0.003	0.62 (0.43–0.88)	0.008

Model 1: crude.

Model 2: adjusted with age, gender, hypertension, diabetes, smoking, ratio of waist to hip, medication.

Model 3.: adjusted with age, gender, hypertension, diabetes, smoking, ratio of waist to hip, medication, lipid, HsCRP, TNF-α.

## Discussion

Our study is the first to show that Chinese CAD patients who take oat fiber were associated with a significantly lower risk of future revascularization and total adverse cardiovascular events compared to the no oat fiber group. In addition, oats intake was found to be associated with reduced serum levels of LDL, decreased TC/HDL ratio, as well as decreased inflammatory markers, suggesting that oats intake may be related to benefits of lipid lowering and reduced inflammation in these patients.

Oats are a rich source of β-glucan which is effective at reducing both total cholesterol and LDL cholesterol^3,11^. It has been established that the consumption of at least 3 g per day of oat β-glucan can achieve a reduction in LDL cholesterol of up to 10% and reduce the risk of CVD by as much as 20%^7^. Therefore, the recommendation guidelines have permitted for cholesterol-lowering effects at this level of β-glucan^12^. However, there is limited evidence of the benefits of oats β-glucan intake for secondary prevention to CAD patients. Our current study is the first to demonstrate the association of oat fiber intake in well-treated CAD patients after coronary intervention. Oat fiber intake was found to be associated with reduced future risk of revascularization and total CV events. In our current study, patients who take oat fiber regularly had lower LDL, lower TGs and lower TC/HDL ratios. There were accumulating evidences which have strongly established the causal role of low-density lipoprotein cholesterol (LDL-C) in atherosclerosis, and very high-risk patients are advised to achieve a target serum LDL level <70 mg/dL^13,14^. Recent secondary prevention trials such as IMPROVE-IT trials^15^ and PCSK9 Inhibition trials which further evaluated lowering LDL-C levels to 30 mg/dL^16^, showed that LDL reduction was significantly associated with reduction in CV risk, and maintained low LDL-C was crucial to prevent the occurrence of poor outcome. In addition to LDL reduction, oat fiber may help to lower blood sugar and improve insulin resistance, especially in those who are overweight and type 2 diabetes^17,18^. Furthermore, our current study showed that oat fiber intake was associated with reduced inflammation markers values and reduced risk of developing poor outcome, supporting the beneficial effect of oat fiber in high-risk patients for 2^nd^ prevention.

Several mechanisms have been proposed to explain the lipid- and glucose-lowering benefits of oat fiber. Oat has been shown to increase the viscosity of the gut digestion, and thereby delay nutrient absorption from the gut^15^. Wood *et al*. reported an inverse linear relationship between peak postprandial blood glucose and insulin increments and viscosity^19^. Oat β-glucan can increase the excretion of cholesterol-rich bile, reducing circulating cholesterol level and Chen *et al*. further demonstrated the antioxidants in oat can act together with vitamin C to prevent LDL from oxdidation, supporting the benefits of oat to the injury of LDL^20^. A recent study reported that oat intake can reduce appetite^21^. In our present study, we showed that oat fiber intake was associated with lower LDL levels and the level of inflammation markers. Oat was shown to reduce the area of atherosclerotic lesions in the aorta, as well as the levels of inflammatory markers fibrinogen and soluble vascular cell adhesion molecule-1 (VCAM-1) in a mouse model, suggesting that oat played a role in inhibiting development of atherosclerotic lesions as well as inflammation^22^. Our data were consistent with a previous study which showed that an oat-enriched diet was associated with a reduced inflammatory status, even in patients with type 2 diabetes already well-controlled by diet and lifestyle alone^23^. In our current study, dietary supplementation with oats was associated with reduced risk of revascularization procedures, but not stroke nor nonfatal MI. This may be because oats intake is mainly associated with cholesterol-lowering and anti-inflammatory effects, and does not significantly impact blood pressure, which is a major risk factor of stoke. There were very few MI events recorded in this study, and the impact of oats intake on occurrence of an MI event needs further investigation after accumulating more cases.

This study had some limitations. Firstly, dietary components are not consumed in isolation, and a higher intake of oat fiber may represent a generally healthier dietary pattern consisting of multiple other interacting food components which are associated with a reduced risk of CVD^24^. Secondly, a wide range of foods contains oat β-glucan. These include rolled oats, whole oat flour, oat bran, bread, muffins, muesli, breakfast cereals, cereal bars, and biscuits. The cholesterol-lowering effect of oats has been shown to depend on viscosity^17^ and variable oat fiber products with differing viscosities could not be standardized in our current study. The Food and Drug Administration has concluded that at doses of 3 g/d oat β-glucan from either approximately 55 g oat bran (minimum 5.5% β-glucan) or 75 g rolled oats (4%) is efficacious in cholesterol lowering^7^. Although oats users were defined according to the above definition in this study, the variable oats products with different viscosities could not be identified in our current study. In addition, the oat use was identified by definition of more than 3 g/d oat β-glucan and maintained during at lest 50% follow-up period. The effect of middle range usage was not evaluated. Furthermore, we did not have the sociodemographic factors information in current study and this important confounding factor was not adjusted. Finally, this is a cohort study from patients with CVD, so the selection bias from a patient cohort can’t be excluded^25^.

In summary, our data showed that oatmeal intake was associated with lower risk of revascularization and total major adverse cardiovascular events in Chinese patients after coronary intervention. Patients taking oats had lower serum levels of LDL, triglycerides, ratio of TC/HDL, and lower levels of inflammatory markers, indicating oat fiber consumption was associated with better lipid control and reduced inflammatory status in these patients.
